# Strategies for rehabilitation management with implants in patients with down syndrome: a scoping review

**DOI:** 10.4317/medoral.27093

**Published:** 2025-05-27

**Authors:** Jessika Dethlefs-Canto, Solange Baeza-Vallejos, Daniela Ormeño-Sepúlveda, Alexis Bustos-Ponce

**Affiliations:** 1 Department of Oral and Maxillofacial Surgery, University of Valparaíso - Valparaíso, Chile; 2 Hospital de Urgencia Asistencia Pública - Santiago, Chile

## Abstract

**Background:**

Down Syndrome (DS), caused by an extra chromosome 21, has a prevalence of 24.7 per 10,000 live births in Chile, the highest in Latin America. Individuals with DS commonly present orofacial and dental anomalies, complicating oral health management. Many depend on removable prostheses, which represent challenges in hygiene, handling, and adaptation. Dental implants are a promising alternative, offering improved stability and functionality. However, successful rehabilitation requires addressing specific anatomical, physiological, and behavioral considerations. This scoping review compiles evidence-based strategies to guide implant treatment in this population.

**Material and Methods:**

Registered on the Open Science Framework (https://osf.io/bstwk/), this review followed the PRISMA-ScR protocol, addressing the question: "Which are the management strategies and survival rates of dental implants in patients with Down Syndrome?" Searches were conducted in Pubmed/MEDLINE, Scopus, Science Direct, Web of Science, and Ebsco databases.

**Results:**

Of 92 studies identified, 7 met inclusion criteria, encompassing 179 implants in DS patients. Anesthesia type varied based on patient cooperation and procedure complexity: general anesthesia for uncooperative patients, local anesthesia for compliant individuals, and sedation for intermediate cases. Delayed loading (3-12 months) yielded better outcomes than immediate loading. Overdentures with locator or bar systems were effective and easier to maintain, while screw-retained fixed prostheses provided stability but required strict hygiene adherence. Clinical success rates varied, with higher success in simple cases and higher failure rates in studies involving multiple implants.

**Conclusions:**

Dental implants, combined with structured behavioral management, improve oral rehabilitation outcomes in DS patients. While sedation or general anesthesia may be required, associated risks must be carefully managed. Delayed implant loading is recommended to minimize osseointegration failures. An interdisciplinary approach, including material selection, caregiver education, and long-term maintenance, is essential for successful, individualized outcomes.

** Key words:**Down syndrome, dental implant.

## Introduction

Down Syndrome (DS) is caused by the presence of an extra chromosome 21, resulting in a series of recognizable clinical characteristics (1). It is the most common cause of intellectual disability of genetic origin, with a prevalence of 1 in 700 live births (2). In Chile, the prevalence of DS is estimated at 26/10,000 births, the highest rate in Latin America (3).

This syndrome presents a wide range of pathologies that affect almost every system in the body, including the cardiovascular, hematological, musculoskeletal, nervous, endocrine, and digestive systems. It is associated with many orofacial and dental alterations, such as hypodontia, malocclusion, bruxism with associated dental wear, reduced vertical dimension, chronic periodontal disease, hypotonic orofacial musculature, reduced salivary flow, and a high incidence of caries. These conditions influence dental treatment, and in many cases, the use of tooth replacements or complete rehabilitations is necessary due to tooth loss or agenesis (4,5).

Most patients with DS successfully use removable dentures, however, some have intellectual disabilities, which can make rehabilitation with removable dentures challenging due to difficulties with hygiene, management, and adaptation. In these cases, fixed dental prostheses provide a rehabilitative alternative, and in some patients, dental implants are the only viable anchorage option (4,6).

Considering the benefits that implant treatment offers as a therapeutic alternative, it is important for professionals to understand the particularities and considerations of these patients. The present review aims to look over the strategies currently used and recommended by professionals in the literature for the management of DS patients with implants, in order to provide considerations that allow dentists to promote the inclusion of individuals with different abilities and their access to the high-quality treatments offered by modern dentistry, especially considering the increase in life expectancy of DS patients over 60 years old.

## Material and Methods

**Figure 1:**
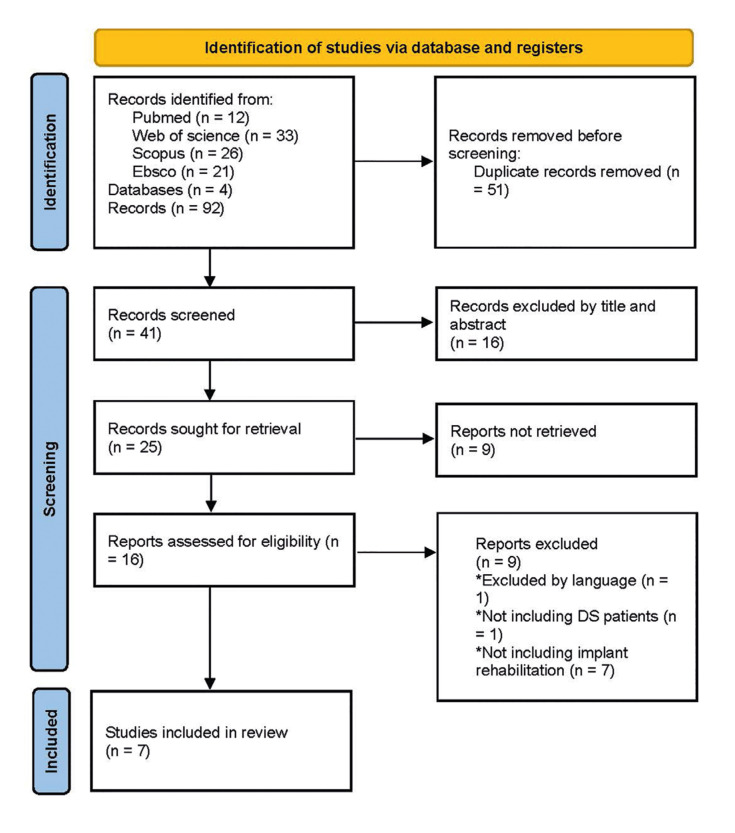
PRISMA Flowchart.

The protocol of the present study was based on the framework of Peters *et al*. according to The Joanna Briggs Institute and is available on the Open Science Framework platform (https://osf.io/kpcm5/). The reporting was based on the PRISMA Extension for Scoping Reviews (PRISMA ScR) (Fig. 1). The PCC question was formulated as follows:

1. People: Down Syndrome Patients

2. Concept: Oral Implant Rehabilitation Management Strategies

3. Context: Oral Rehabilitation in disabled patients

According to the research question:

What are the management strategies and survival rates of dental implants in patients with Down syndrome?

The researchers (J.D.; D.O.) conducted a search strategy in parallel and independently on the platforms PubMed, Scopus, Science Direct, and Web of Science. The search terms in titles and abstracts included the terms (dental implant[MeSH Terms]) and ("down syndrome"[MeSH Terms]) using the boolean connector "AND." Obtaining 7 articles in total. Last search was conducted on October 2024.

Inclusion Criteria:

1. Studies in English.

2. Full-text studies published between 2014 and 2024.

3. Case reports.

4. Case series studies (retrospective and cohort) and clinical trials.

5. Studies conducted in patients with down syndrome rehabilitated with dental implants.

Exclusion Criteria:

6. Book chapters.

7. Letters to the editor.

8. Systematic reviews.

## Results

- Use of anaesthesia

The use of anaesthesia in patients DS depends on their level of cooperation and the complexity of the procedure. General anaesthesia is common in uncooperative patients or in complex clinical conditions (7,8). On the other hand, local anaesthesia is effective for cooperative patients and simpler procedures (9,10). Sedation is used as an intermediate approach in mixed cases, providing adequate control in specific situations.

- Loading periods

**Figure 2:**
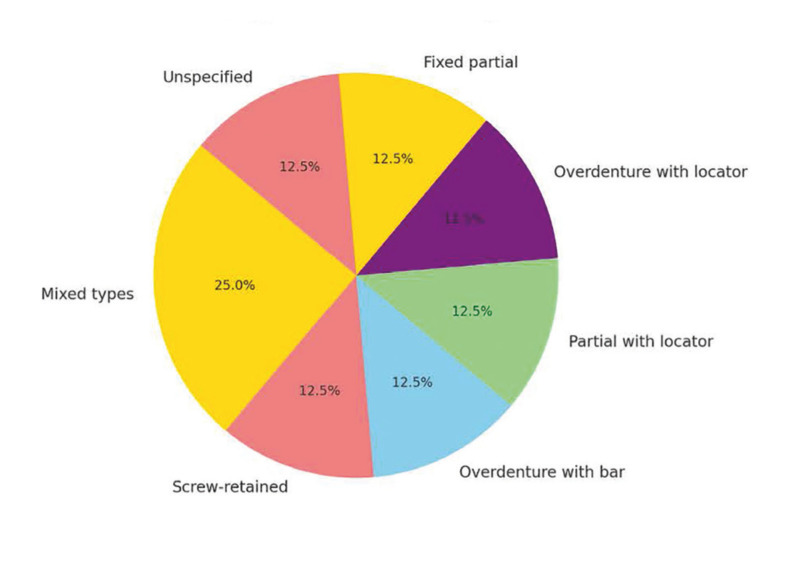
Success Rates by Loading Period.

Studies show that delayed loading (between 3 and 12 months) offers better results than immediate loading (9,11) (Fig. 2).

The study by Comparin *et al*. (2022) highlights a higher failure rate for immediately loaded implants, underlining the importance of allowing adequate time for osseointegration (7).

- Rehabilitation and prosthetics

**Figure 3:**
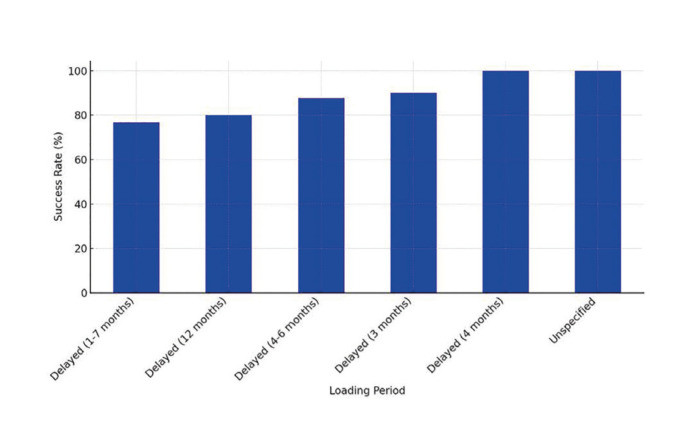
Prosthetic Type Distribution Among Studies.

Overdentures with locator or bar systems were found to be successful and easier to maintain for both patients and their caregivers. Screw-retained fixed partial dentures offered greater stability, but required rigorous adherence to oral hygiene practices (7,11,12) (Fig. 3).

- Hygiene strategies and behavioural management

Hygiene education was a key element in preventing peri-implantitis, according to several studies. In addition, the use of behavioural techniques, such as ritualised routines, improved patient cooperation during treatment (9,11).

- Clinical outcomes

**Figure 4:**
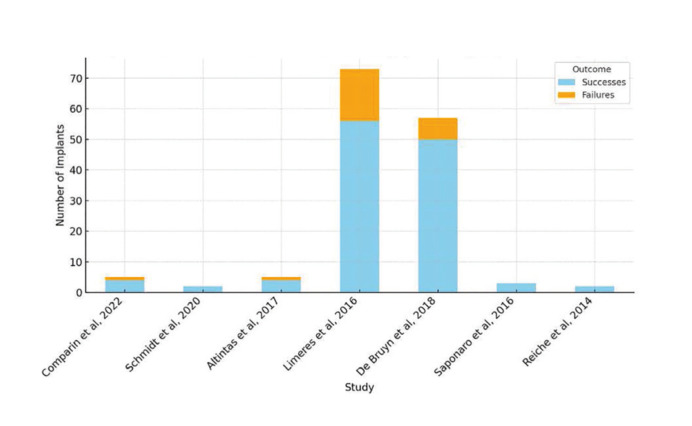
Implant Success and Failure Rates Per Study.

Success rates varied significantly according to case complexity and patient compliance (Fig. 4). The highest success rates were reported by Saponaro *et al*. (2016) and Schmidt *et al*. (2020), reaching 100% (10,11). On the other hand, studies with higher numbers of implants placed, such as Limeres *et al*. (2016), showed higher failure rates (2) (Table 1).

## Discussion

All the studies refer to implants as a treatment with potential for success and survival. The literature reports success rates of 74-85% (7). Shariq Najeeb *et al*. (2017) compare the success rate of implants over 10 years, reporting a 96% success rate in the general population. However, in their systematic review, patients with DS show a 26% failure rate at six years, suggesting a higher risk of implant loss in this population. The authors associate these results with studies indicating that patients with DS may have reduced bone density. Therefore, they recommend coating the surface of the implants to improve osseointegration outcomes, which could benefit patients with DS (13). Given that these patients often struggle with removable prostheses due to difficulties in placement, removal, hygiene, limited comfort due to increased lingual pressure, muscle hypotonia, xerostomia, bruxism, and palate characteristics, implants are an option that should be considered and presented as a possibility that not only restores function but also preserves bone (7,12).

The reviewed studies emphasize the importance of providing hygiene instructions to both patients and their caregivers, as well as conducting maintenance sessions to ensure proper hygiene practices. Shariq Najeeb *et al*. (2017) highlight poor oral hygiene in DS patients, which contributes to implant failure and increased susceptibility to peri-implantitis (13). Corcuera *et al*. (2016) conducted a study comparing survival rates and marginal bone loss (MBL) in DS patients, patients with cerebral palsy (CP), and those without systemic conditions. They found that the first two groups had higher implant failure rates and MBL, with DS patients showing a greater prevalence of both indicators compared to CP patients. Considering that both conditions are characterized by poor oral hygiene and parafunctional habits, the results suggest that immune deficiencies may play a larger role in implant success. However, the study was conducted on a small population, so larger studies are needed to confirm this correlation (14). These findings are supported by other studies indicating that the etiology of periodontal disease in DS patients differs from those without systemic conditions, suggesting that immune deficiencies contribute to the rapid progression of this disease (12).

Regarding management strategies for implant placement and connection surgeries, most studies highlight the use of general anesthesia. Rehabilitation in the reviewed works was achieved under local anesthesia with protocolized behavioral management techniques. For general anesthesia, prior knowledge of the risks is essential. In DS patients, anesthesiologists must consider occipito-atlanto-axial instability, which occurs in approximately 15% of cases. This instability, caused by joint laxity, skeletal abnormalities, or both, can lead to neurological conditions, including quadriplegia (15). Another technique used in the reviewed literature was intravenous sedation, applied in one study for connection surgery and in another for implant surgery. It is important to note that DS patients are more susceptible to decreases in peripheral oxygen saturation after intravenous sedation (16). Yoshikawa *et al*. determined that DS patients posed the greatest risk of poor sedation outcomes compared to CP patients and those with intellectual disabilities. Poor oxygenation during sedation is associated with sleep apnea and airway obstruction due to macroglossia, necessitating careful monitoring of these patients (2). Whenever possible, dental treatment should be performed with the patient awake using local anesthesia. However, the decision on the type of anesthesia for each treatment should be individualized, considering the patient’s ability to cooperate (11).

Regarding clinical management of awake patients in the dental chair, only two studies reported strategies for addressing DS patients. Schmidt *et al*. performed procedures using protocolized behavioral management techniques. These techniques were considered promising due to DS patients need for consistent routines and familiar procedures, which are frequently described as characteristic behaviors in this population. Techniques included voice control, verbal explanations, and presenting materials and instruments (e.g., impression materials and trays) to the patient. Patients were allowed to touch the materials and trays, continuing with these techniques in subsequent sessions (11).

Implants in DS patients were mostly loaded late, varying from 1 to 12 months post-surgery. Not all failed implants were immediate-load, but all attempted immediate-load implants failed. Most implant failures in DS patients occurred during the osseointegration phase. This requires excluding variables such as the time between implant placement and loading, type of prosthetic rehabilitation, tongue pressure on implants, and other factors described in patients with intellectual disabilities, including poor oral hygiene and parafunctional habits like bruxism (2). While some studies have documented immediate loading in DS patients, the data suggest that bruxism may contribute to early failure of immediate-load implants, leading to recommendations to avoid this approach. However, most failures reported by Posse *et al*. occurred before loading, suggesting other factors at play (13). Baus-Dominguez *et al*. (2019) found a relationship between MT1 and MT2 genes and implant failure or peri-implantitis. Expression of these genes plays a crucial role in early osteogenic cell differentiation. The authors observed lower expression of these genes in DS patients with failed osseointegration or peri-implantitis compared to those without such issues. This is attributed to reduced metallothioneins, which are critical for antioxidant activity and cell differentiation in new bone formation (4).

In addition, in the case of patients with DS, there are several other aspects that need to be taken into account in the decision process for dental implants. One of these is that the extent of the intellectual disability associated with DS may differ considerably between individuals. This can pose a further challenge in the treatment of these patients. Another important consideration is that, among persons with disabilities, individuals with (12).

This heightened anxiety demands great flexibility on the treating clinician’s part. It is also the reason why it may still be necessary to carry out certain treatment steps, like the insertion of dental implants in combination with other oral surgical measures, under sedation or general anesthesia in patients with Down syndrome, even if the clinician is experienced in behavior management. reclining position on the dental chair, may be found in Windman’s false-alarm theory. According to this theory, in some individuals, the limbic system, which is involved in the pre-attentive processing of stimuli and, thus, also functions as a biologic alarm system, is insufficiently inhibited by higher-order cortical structures. In consequence, the limbic system registers fear too frequently. This also happens in objectively unthreatening “false alarm” situations. Affected individuals may thus perceive a threat and respond with fear even in the absence of objectively threatening stimuli (16).

The degree of intellectual disability in Down syndrome patients is variable, ranging from mild (IQ: 50 to 70) to moderate (IQ: 35 to 50) to severe (IQ: 20 to 35) (12).

The patient had mild mental impairment but was very enthusiastic about dental implants and reported that she wished to be attractive with her new prosthesis (12). In the present report, dental implants were placed under local anesthesia, because the patient had a mild mental disability and was very cooperative during the examinations.

This study has several limitations, including a small sample size and the lack of long-term follow-up data to assess the stability and success of dental implants in Down syndrome (DS) patients. The heterogeneity of the included studies and the limited representation of individuals with severe intellectual disabilities restrict the generalizability of the findings. Additionally, there is a need for research addressing the role of genetic factors, such as the expression of MT1 and MT2 genes, in implant outcomes. Future studies should focus on larger, multicenter cohorts, explore long-term outcomes, and investigate personalized treatment strategies tailored to the varying degrees of intellectual disability. Furthermore, incorporating advanced technologies, evaluating caregiver support, and conducting comparative analyses of implant loading protocols could improve outcomes and enhance the quality of care for this population.

## Conclusions

Dental implants are a viable option for patients with Down syndrome (DS), although they have higher failure rates (26% over six years) due to low bone density, poor hygiene and parafunctional habits. Delayed loading has shown better results than immediate loading, and overdentures with locator or bar systems are easier to maintain than screw-retained fixed prostheses, which require strict hygiene.

Clinical management should be adapted to the degree of intellectual disability and consider anaesthetic risks such as occipito-atlanto-axial instability. Hygiene education and caregiver support are essential to prevent complications such as peri-implantitis. An interdisciplinary and personalised approach ensures better functional outcomes and a better quality of life for these patients.

## Figures and Tables

**Table 1:** Results.

Author	Anesthesia	Loading	Rehabilitation	Strategies	Clinical Outcomes
Reiche et al, 2014	-	-	-	Unspecified	Two successful implants
Saponaro et al, 2016	Implant Surgery: Local anesthesiaConnection Surgery: Local anesthesiaRehabilitation: Local anesthesia	4 months	Screw-retained fixed partial prosthesis	The patient exhibited sufficient maturity and understanding to effectively cooperate during all surgical and prosthetic phases of her treatment. However, instructions and reinforcement for the maintenance and success of her treatment were emphasized to all parties at the end of each appointment 2 stage protocol	Three successful implants
Limeres et al, 2016	Implant Surgery: - General anesthesia: 17 patients - Deep sedation: 4 patients - Local anesthesia: 4 patients Connection Surgery: Not specified. Rehabilitation: Not specified.	1-7 months after surgery	Single crown: 13 patients Fixed partial prosthesis: 5 patients Overdenture: 2 patients Full fixed lower prosthesis: 3 patients Single crown + fixed partial prosthesis: 2 patients	Unespecified	73 implants placed17 failures (all failed prior to loading)
Altintas et al, 2017	Implant Surgery: Local anesthesia Connection Surgery: Not specified Rehabilitation: Not specified	Delayed loading after 3 months	Overdenture with locator	Implant Surgery under local anesthesiaHygiene instructions provided to the patient and caregivers.	Five implants, one lost. Overdentures
De Bruyn et al, 2018	Implant Surgery: General anesthesia Connection Surgery: General anesthesia Rehabilitation: Some installed during connection surgery, some with local anesthesia, some without general anesthesia.	4-6 months	Single crown: 1 patient Full fixed upper prosthesis: 2 patients Full fixed lower prosthesis: 2 patients Fixed partial prosthesis: 1 patient	Implant and connection surgery under general anesthesia. General anesthesia for rehabilitation depending on the patient's cooperation. Two-stage protocol to avoid excessive tongue pressure.	57 implants 7 failures
Schmidt et al 2020	Implant Surgery: General anesthesia Connection Surgery: General anesthesia Prosthetic Rehabilitation: Local anesthesia	Delayed loading after 3 months	Overdenture with bar	Ritualized behavior management techniques. Hygiene instructions provided to the patient and caregivers. Postural adaptation adjustments to the patient's position during clinical care	Two successful implants with mandibular overdenture.A bar system was used for the overdenture due to its ease of positioning.
Comparin et al 2022	Implant Surgery: General anesthesia in a hospital setting. Connection Surgery: Intravenous sedation in the dental office. Prosthetic Rehabilitation: Local anesthesia.	Delayed loading after 12 months	Screw-retained (plural)	Use of PRF and hygiene instructions provided to both the patient and the caregiver, with frequent follow-ups	Five implants placed, one failure (the failure occurred with immediate loading).
